# Enrichment of hematopoietic stem/progenitor cells in the zebrafish kidney

**DOI:** 10.1038/s41598-019-50672-5

**Published:** 2019-10-02

**Authors:** Isao Kobayashi, Mao Kondo, Shiori Yamamori, Jingjing Kobayashi-Sun, Makoto Taniguchi, Kaori Kanemaru, Fumihiko Katakura, David Traver

**Affiliations:** 1 Faculty of Biological Science and Technology, Institute of Science and Engineering, Kanazawa University, Kanazawa, Ishikawa Japan; 2 Division of Life Sciences, Graduate School of Natural Science and Technology, Kanazawa University, Kanazawa, Ishikawa Japan; 3 Department of Life Science, Medical Research Institute, Kanazawa Medical University, Uchinada, Ishikawa Japan; 4 Department of Applied Biological Science, Faculty of Science and Technology, Tokyo University of Science, Noda, Chiba Japan; 5 Laboratory of Comparative Immunology, Department of Veterinary Medicine, Nihon University, Fujisawa, Kanagawa Japan; 6 Department of Cellular and Molecular Medicine, University of California at San Diego, La Jolla, CA USA

**Keywords:** Haematopoietic stem cells, Stem-cell differentiation

## Abstract

Hematopoietic stem cells (HSCs) maintain the entire blood system throughout life and are utilized in therapeutic approaches for blood diseases. Prospective isolation of highly purified HSCs is crucial to understand the molecular mechanisms underlying regulation of HSCs. The zebrafish is an elegant genetic model for the study of hematopoiesis due to its many unique advantages. It has not yet been possible, however, to purify HSCs in adult zebrafish due to a lack of specific HSC markers. Here we show the enrichment of zebrafish HSCs by a combination of two HSC-related transgenes, *gata2a:GFP* and *runx1:mCherry*. The double-positive fraction of *gata2a:GFP* and *runx1:mCherry* (*gata2a*^+^ *runx1*^+^) was detected at approximately 0.16% in the kidney, the main hematopoietic organ in teleosts. Transcriptome analysis revealed that *gata2a*^+^ *runx1*^+^ cells showed typical molecular signatures of HSCs, including upregulation of *gata2b*, *gfi1aa*, *runx1t1*, *pbx1b*, and *meis1b*. Transplantation assays demonstrated that long-term repopulating HSCs were highly enriched within the *gata2a*^+^ *runx1*^+^ fraction. In contrast, colony-forming assays showed that *gata2a*^−^ *runx1*^+^ cells abundantly contain erythroid- and/or myeloid-primed progenitors. Thus, our purification method of HSCs in the zebrafish kidney is useful to identify molecular cues needed to regulate self-renewal and differentiation of HSCs.

## Introduction

Hematopoietic stem cells (HSCs) are self-renewing multipotent cells that can generate all types of blood cells over the lifetime of an individual and can be used therapeutically to treat hematopoietic diseases^1^. In the adult, most HSCs present in bone marrow are quiescent and divide rarely under homeostatic conditions. HSCs produce a heterogeneous pool of hematopoietic progenitor cells (HPCs), which have limited or no self-renewal ability, but rapidly proliferate and differentiate to satisfy the requirements for new mature blood cells^2,3^. Although the frequency of HSCs is extremely rare in bone marrow, HSC potential can be evaluated by transplantation assays, whereby the relative hematopoietic reconstitution activity of co-transplanted donor and competitor cells are compared in a recipient^4^. Purification of HSCs from murine and human bone marrow has been facilitated via transplantation assays using combinations of multiple cell-surface markers^5–9^. Studies in mice revealed that a single CD150^+^ CD34^−^ c-kit^+^ Sca-1^+^ Lineage-marker^−^ cell in the bone marrow showed long-term and multilineage hematopoietic reconstitution following transplantation^10,11^. Prospective isolation of highly purified HSCs thus elucidated many aspects of HSC biology, including self-renewal, differentiation, and HSC niches.

The zebrafish is an excellent model for the study of HSCs due to its many unique advantages. Many valuable tools and experimental methods have been established for the study of hematopoietic cells in zebrafish (e.g. transgenic/mutant animals, transplantation assays, cell culture assays, etc.)^12,13^. Moreover, genome-editing technology based on the CRISPR/Cas9 system has further facilitated rapid and scalable reverse genetic approaches in zebrafish^14–16^. Hematopoiesis is highly conserved between mammals and teleost fish at both the cellular and molecular level, while the sites of adult hematopoiesis have shifted during the evolution. The major hematopoietic organ in teleost fish is the kidney where various stages of hematopoietic cells and mature blood cells are observed in the interstitial tissue, the so-called “kidney marrow”^17–20^. Although the zebrafish kidney is of great importance to identify evolutionarily conserved regulators of HSCs, little is known regarding which molecules are involved in the maintenance and self-renewal of HSCs in the kidney. This is due in part to the lack of robust methods to purify HSCs in the zebrafish kidney.

The enrichment of HSCs from the kidney has been shown previously in zebrafish. A previous method using the light scatter profile of whole kidney marrow cells (WKMCs) by flow cytometric (FCM) analysis demonstrated that HSCs are present within the forward scatter (FSC)^low^ side scatter (SSC)^low^ “lymphoid” fraction^17^. The Hoechst dye efflux activity of HSCs is highly conserved amongst vertebrates^21^, and an enriched HSC fraction has been isolated by sorting of “side population” (SP) cells in the kidney^22,23^. More recently, two groups demonstrated using a transgenic zebrafish line that HSCs are present in the *cd41:GFP*^low^ fraction or *runx1:mCherry*^+^ fraction in the kidney^24,25^. These transgenic animals are also utilized to visualize hematopoietic stem/progenitor cells (HSPCs) in developmental stages^25–27^. The expression of *cd41:GFP* and *runx1:mCherry* as well as the SP phenotype is, however, not specific to HSCs, indicating that a combination of multiple markers is required to further purify HSCs in the zebrafish kidney, as has been proven in mammalian bone marrow^5–9^.

In the present study, we combined two transgenic markers of putative HSCs, *gata2a:GFP* and *runx1:mCherry*, and found that zebrafish HSCs are highly enriched in the double-positive fraction (*gata2a:GFP*^+^ *runx1:mCherry*^+^, hereafter denoted as *gata2a*^+^ *runx1*^+^) in the kidney. Transcriptome analysis of three distinct hematopoietic cell populations, *gata2a*^+^ *runx1*^+^, *gata2a*^−^ *runx1*^+^, and *gata2a*^+^ *runx1*^−^ cells, revealed that *gata2a*^+^ *runx1*^+^ cells displayed typical molecular hallmarks of HSCs. In contrast, *gata2a*^−^ *runx1*^+^ showed expression signatures of erythroid and myeloid cells, whereas *gata2a*^+^ *runx1*^−^ cells showed those of lymphoid and myeloid cells. Transplantation assays demonstrated that *gata2a*^+^ *runx1*^+^ cells possessed the high level of long-term and multilineage hematopoietic reconstitution activity. Thus, we provide evidence that combined *gata2a:GFP* and *runx1:mCherry* expression is a useful method to purify HSCs from the zebrafish kidney.

## Results

### Isolation of HSPCs using *gata2a:GFP; runx1:mCherry* double-transgenic zebrafish

**Figure 1:**
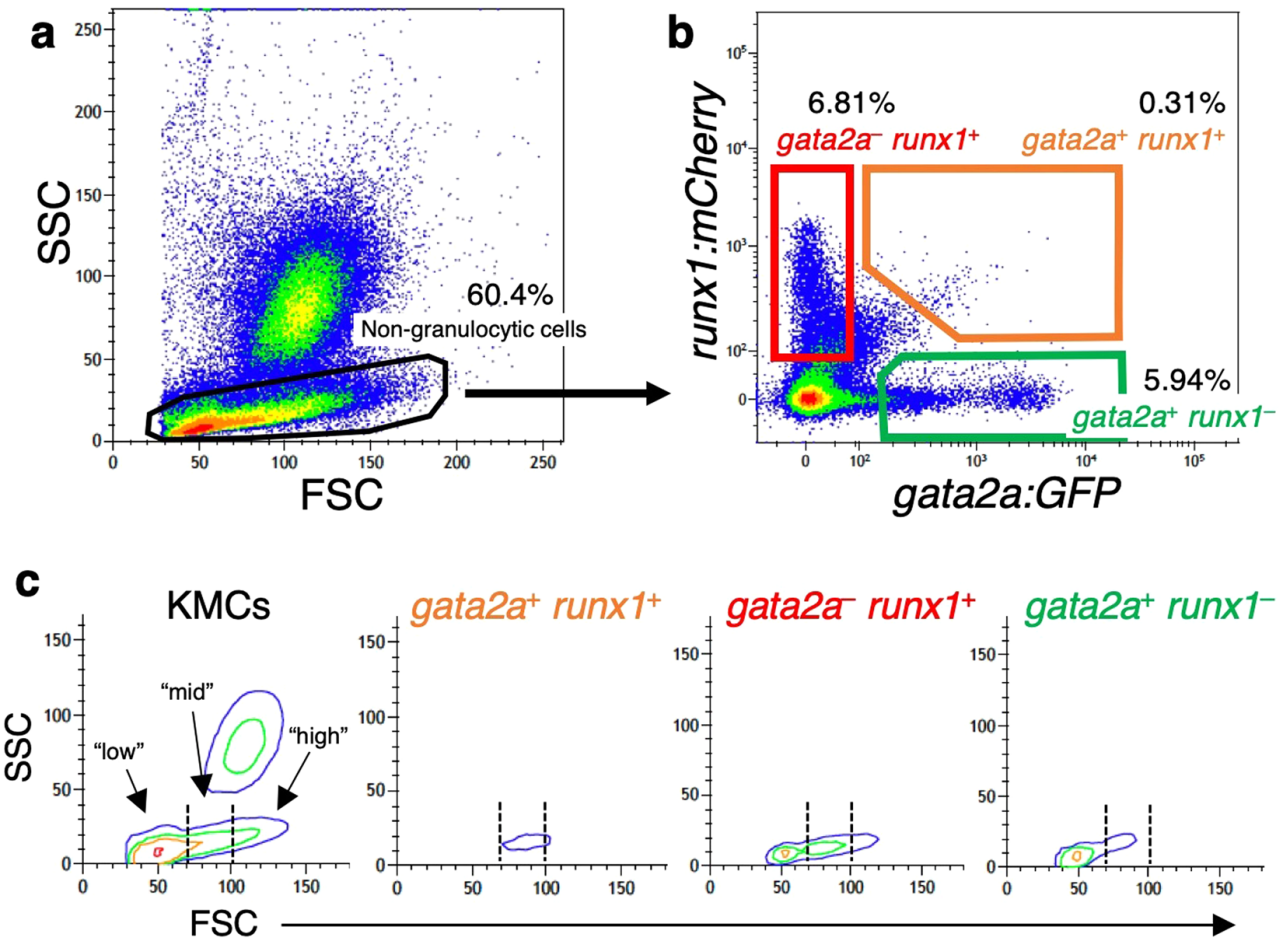
Isolation of HSPCs from the zebrafish kidney. FCM analysis of KMCs was performed in a *gata2a:GFP*; *runx1:mCherry* double-transgenic zebrafish. (**a**) A scatter profile of KMCs. The SSC^low^ non-granulocytic cell fraction is gated. (**b**) SSC^low^ cells are subdivided into three distinct hematopoietic populations, *gata2a*^+^ *runx1*^+^ (orange gate), *gata2a*^−^ *runx1*^+^ (red gate), and *gata2a*^+^ *runx1*^−^ (green gate). (**c**) Contour plot of FSC vs. SSC in KMCs, *gata2a*^+^ *runx1*^+^, *gata2a*^−^ *runx1*^+^, and *gata2a*^+^ *runx1*^−^ cells. Dotted lines separate the “low”, “mid”, and “high” intensity of FSC.

In order to purify HSCs from the adult kidney, we utilized *gata2a* (*GATA binding protein 2a*) as an HSC marker, of which expression in HSCs and vascular endothelial cells is well-conserved amongst vertebrates^23,28–30^. A zebrafish *gata2a:GFP* line expresses GFP in a variety of hematopoietic cells and vascular endothelial cells^28^. We combined the *gata2a:GFP* line with the *runx1:mCherry* line, which expresses mCherry under control of the mouse *Runx1* (*Runt-related transcription factor 1*) +23 enhancer^25^. FCM analysis of kidney marrow cells (KMCs), which contain hematopoietic cells and mature blood cells excluding erythrocytes, showed that the majority of *gata2a:GFP*^+^ cells and *runx1:mCherry*^+^ cells within the SSC^low^ fraction (non-granulocytic cells) did not overlap, whereas only 0.31% of SSC^low^ cells were found within the *gata2a*^+^ *runx1*^+^ population (0.16 ± 0.06% in total KMCs, n = 23, ± s.d.) (Fig. 1a,b). HSCs have been shown to be present in the FSC^low^ SSC^low^ “lymphoid” fraction by FCM analysis in the zebrafish kidney^17^. As shown in Fig. 1c, we subdivided SSC^low^ cells into three distinct fractions based on the intensity of FSC, “low” (in the range of 30 to 70), “mid” (in the range of 70 to 100), and “high” (>100) fraction. Unexpectedly, most *gata2a*^+^ *runx1*^+^ cells showed the “mid” intensity of FSC. In contrast, *gata2a*^−^ *runx1*^+^ cells showed the “low” to “high” intensity, and *gata2a*^+^ *runx1*^−^ cells mainly showed the “low” intensity of FSC (Fig. 1c).

**Figure 2:**
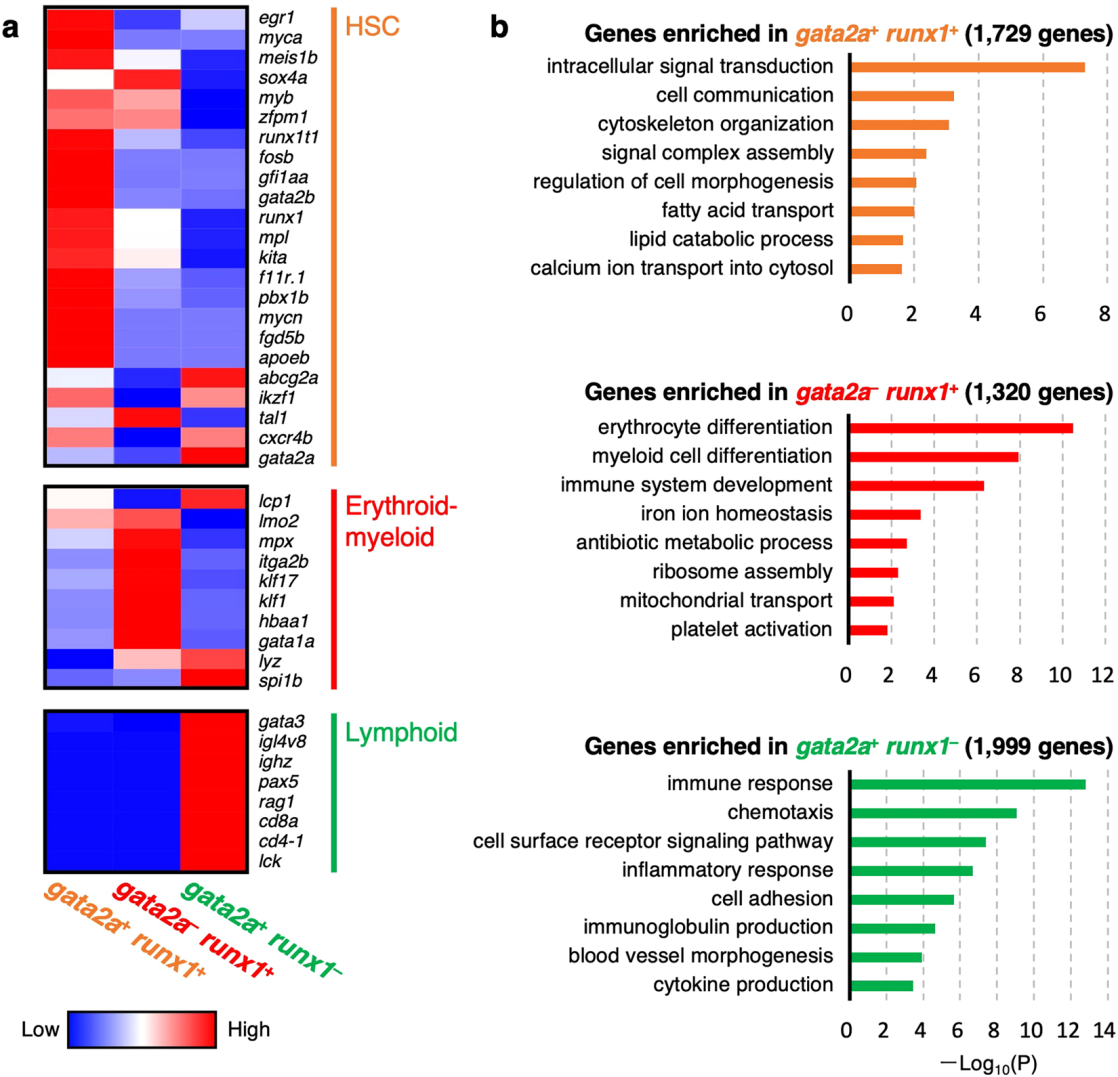
Transcriptome analysis of three distinct hematopoietic populations. (**a**) Hierarchical clustering of selected HSC- (upper), erythroid-myeloid- (middle), and lymphoid-related genes (lower). (**b**) Gene ontology enrichment analysis of highly expressed genes in *gata2a*^+^ *runx1*^+^ (upper), *gata2a*^−^ *runx1*^+^ (middle), or *gata2a*^+^ *runx1*^−^ cells (lower).

**Figure 3:**
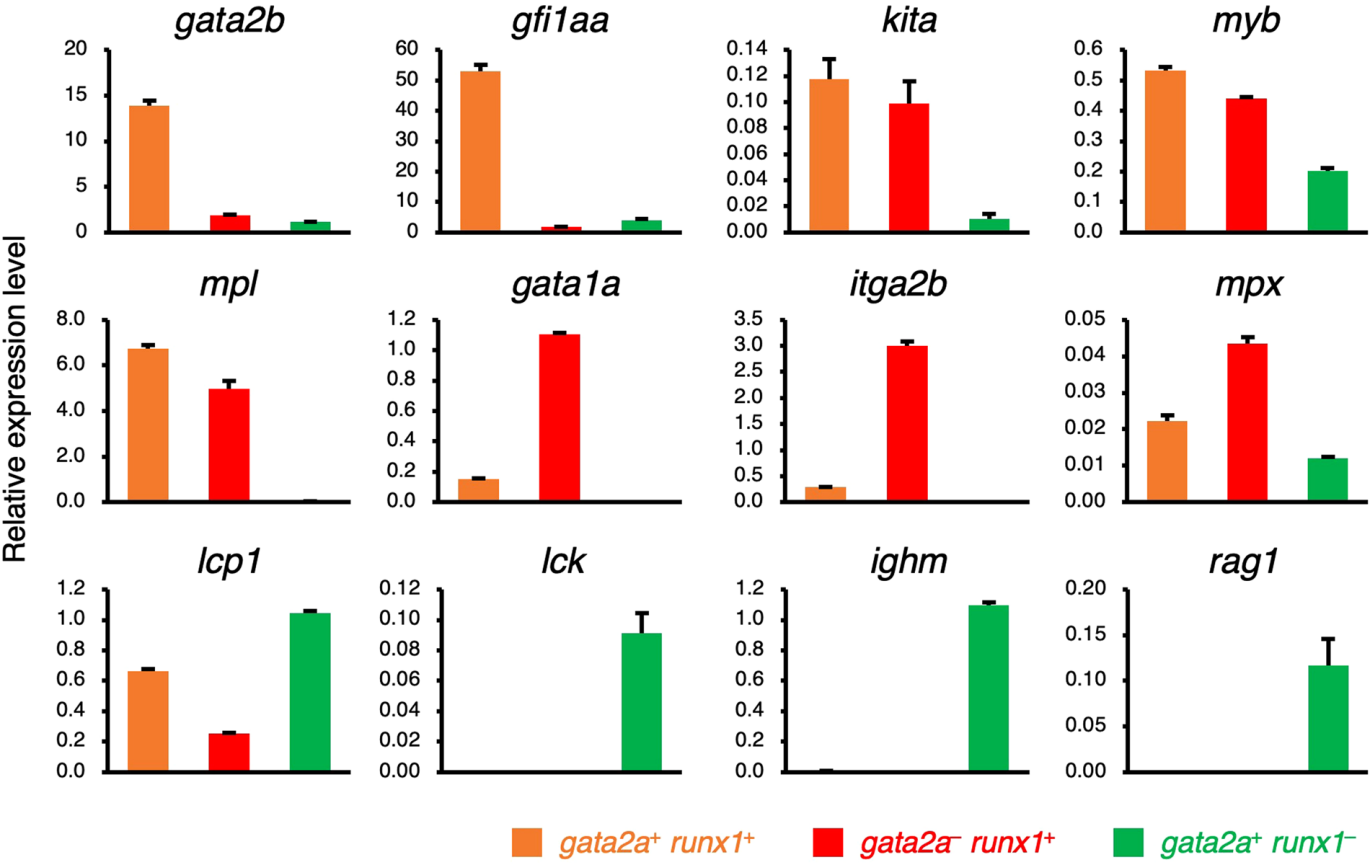
Expression of hematopoietic marker genes in HSPC populations. Relative expression levels of hematopoietic marker genes are shown. Orange, red, and green bars denote *gata2a*^+^ *runx1*^+^, *gata2a*^−^ *runx1*^+^, and *gata2a*^+^ *runx1*^−^ cells, respectively. The expression level in the kidney tissue is shown as 1.0 in each panel. Error bars, s.d.

To characterize hematopoietic cell populations in the kidney, RNA-seq analysis was performed on three distinct cell populations within the SSC^low^ fraction, *gata2a*^+^ *runx1*^+^, *gata2a*^−^ *runx1*^+^, and *gata2a*^+^ *runx1*^−^. As shown in Fig. 2a, HSC-related genes, such as *gata2b* (*GATA binding protein 2b*), *gfi1aa* (*growth factor independent 1A*), *runx1t1* (*runt-related transcription factor 1; translocated to, 1*), *pbx1b* (*pre-B-cell leukemia homeobox 1b*), *fgd5b* (*FYVE, RhoGEF and PH domain containing 5b*), *meis1b* (*Meis homeobox 1b*), *myca* (*MYC proto-oncogene, bHLH transcription factor a*), *apoeb* (*apolipoprotein Eb*), and *egr1* (*early growth response 1*), were highly enriched in *gata2a*^+^ *runx1*^+^ cells. In contrast, erythroid and thrombocyte marker genes, such as *gata1a* (*GATA binding protein 1a*), *klf1* (*Kruppel-like factor 1*), *hbaa1* (*hemoglobin, alpha adult 1*), and *itga2b* (*integrin, alpha 2b*, also known as *cd41*), were strongly expressed in *gata2a*^−^ *runx1*^+^ cells, whereas myeloid marker genes, such as *mpx* (*myeloid-specific peroxidase*), *lyz* (*lysozyme*), *lcp1* (*lymphocyte cytosolic protein 1*, also known as *l-plastin*), and *spi1b* (*Spi-1 proto-oncogene b*, also known as *pu.1*), were expressed in both *gata2a*^−^ *runx1*^+^ cells and *gata2a*^+^ *runx1*^−^ cells. Lymphoid marker genes, such as *rag1* (*recombination activating gene 1*), *cd8a*, *cd4-1*, *lck* (*Lymphocyte-specific protein-tyrosine kinase*), and *ighz* (*immunoglobulin heavy constant zeta*), were detected only in the *gata2a*^+^ *runx1*^−^ fraction, whereas a part of lymphoid marker genes, such as *tcra* (*t cell receptor alpha*), *tcrd* (*t cell receptor delta*), *mhc2dab* (*major histocompatibility complex class II DAB gene*), and *mhc2dbb* (*major histocompatibility complex class II DBB gene*), were undetected in *gata2a*^+^ *runx1*^−^ cells or within other fractions (Supplementary Table S1). To further characterize these hematopoietic cell populations, gene ontology enrichment analysis was performed. We found that genes involved in “intracellular signal transduction” and “cell communication” were significantly enriched in *gata2a*^+^ *runx1*^+^ cells, suggesting that these cells actively communicate with environmental niche cells. In contrast, genes involved in “erythrocyte differentiation” and “myeloid cell differentiation” are enriched in *gata2a*^−^ *runx1*^+^ cells, whereas genes involved in “immune response” and “chemotaxis” are enriched in *gata2a*^+^ *runx1*^−^ cells (Fig. 2b). Quantitative PCR (qPCR) analysis also showed that HSC-related genes, *gata2b* and *gfi1aa*, were highly expressed in *gata2a*^+^ *runx1*^+^ cells, whereas the expression of *kita* (*KIT proto-oncogene, receptor tyrosine kinase a*), *myb* (*v-myb avian myeloblastosis viral oncogene homolog*), and *mpl* (*MPL proto-oncogene, thrombopoietin receptor*) was detected in both *gata2a*^+^ *runx1*^+^ cells and *gata2a*^−^ *runx1*^+^ cells. In contrast, erythroid and thrombocyte marker genes, *gata1a* and *itga2b*, were strongly expressed in *gata2a*^−^ *runx1*^+^ cells, and lymphoid marker genes, *lck*, *ighm* (*immunoglobulin heavy constant mu*), and *rag1*, were expressed only in *gata2a*^+^ *runx1*^−^ cells. A neutrophil marker, *mpx*, and macrophage marker, *lcp1*, were predominantly expressed in *gata2a*^−^ *runx1*^+^ cells and *gata2a*^+^ *runx1*^−^ cells, respectively (Fig. 3). These expression patterns in qPCR analysis were consistent with those in RNA-seq analysis, confirming the reliability and validity of our transcriptome analysis. Collectively, these data suggest that *gata2a*^+^ *runx1*^+^ cells have typical molecular hallmarks of HSCs, whereas *gata2a*^−^ *runx1*^+^ show expression signatures of erythroid and myeloid cells and *gata2a*^+^ *runx1*^−^ cells show those of lymphoid and myeloid cells.

### *gata2a*^+^*runx1*^+^ cells possess long-term and multilineage hematopoietic reconstitution activity

**Figure 4:**
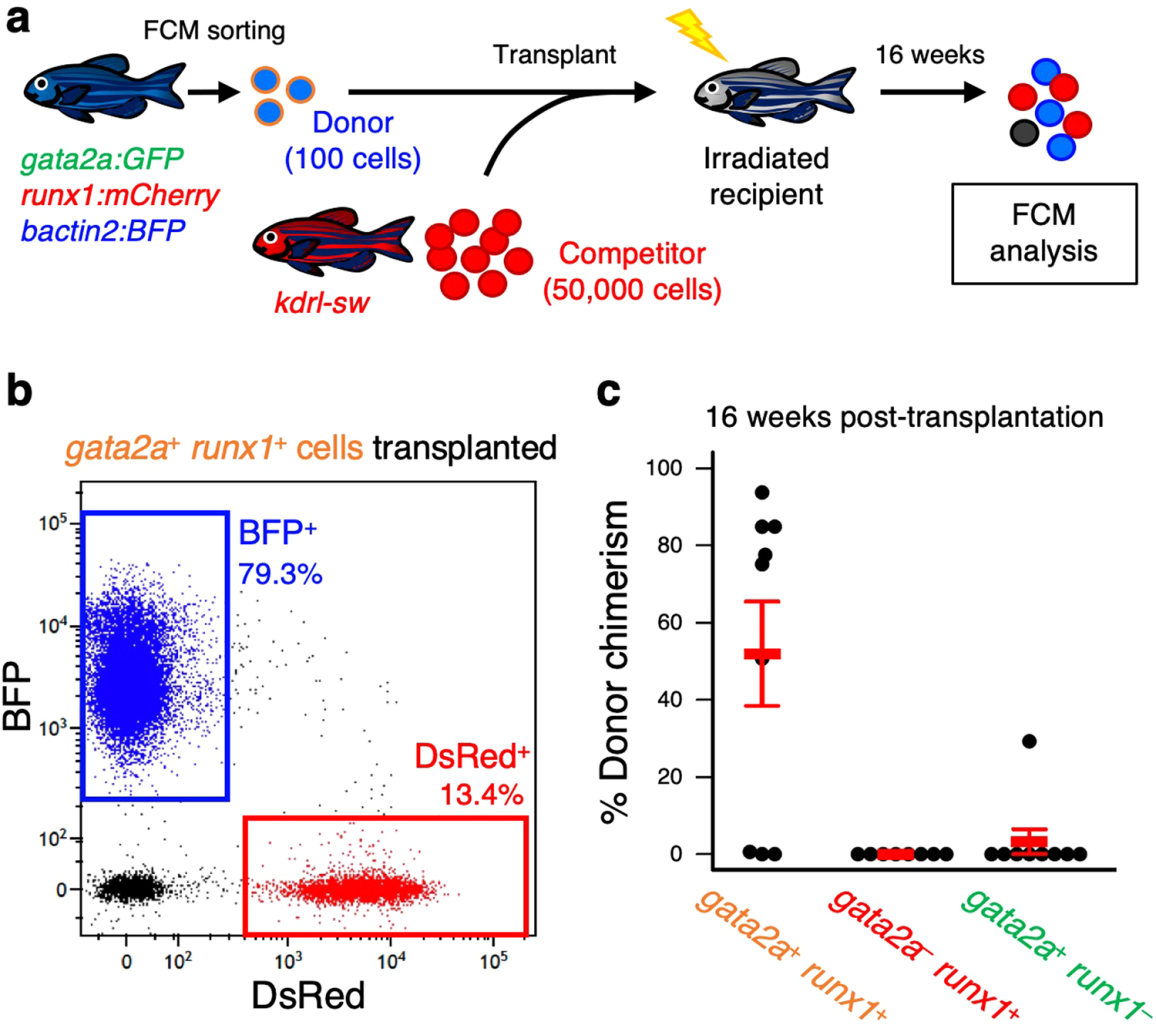
Transplantation assays of HSPC populations. (**a**) Experimental procedure of a competitive repopulation assay. One hundred BFP-labeled donor cells were co-transplanted with 50,000 of DsRed-labeled KMCs (competitors) into a sublethally irradiated recipient animal. At 16 wpt, donor chimerism was determined by the percentage of BFP^+^ cells within the total BFP^+^ and DsRed^+^ cells in each recipient. (**b**) Representative result of FCM analysis in a recipient transplanted with *gata2a*^+^ *runx1*^+^ cells. (**c**) Percentage of donor chimerism in each recipient group (mean ± s.e.m).

**Figure 5:**
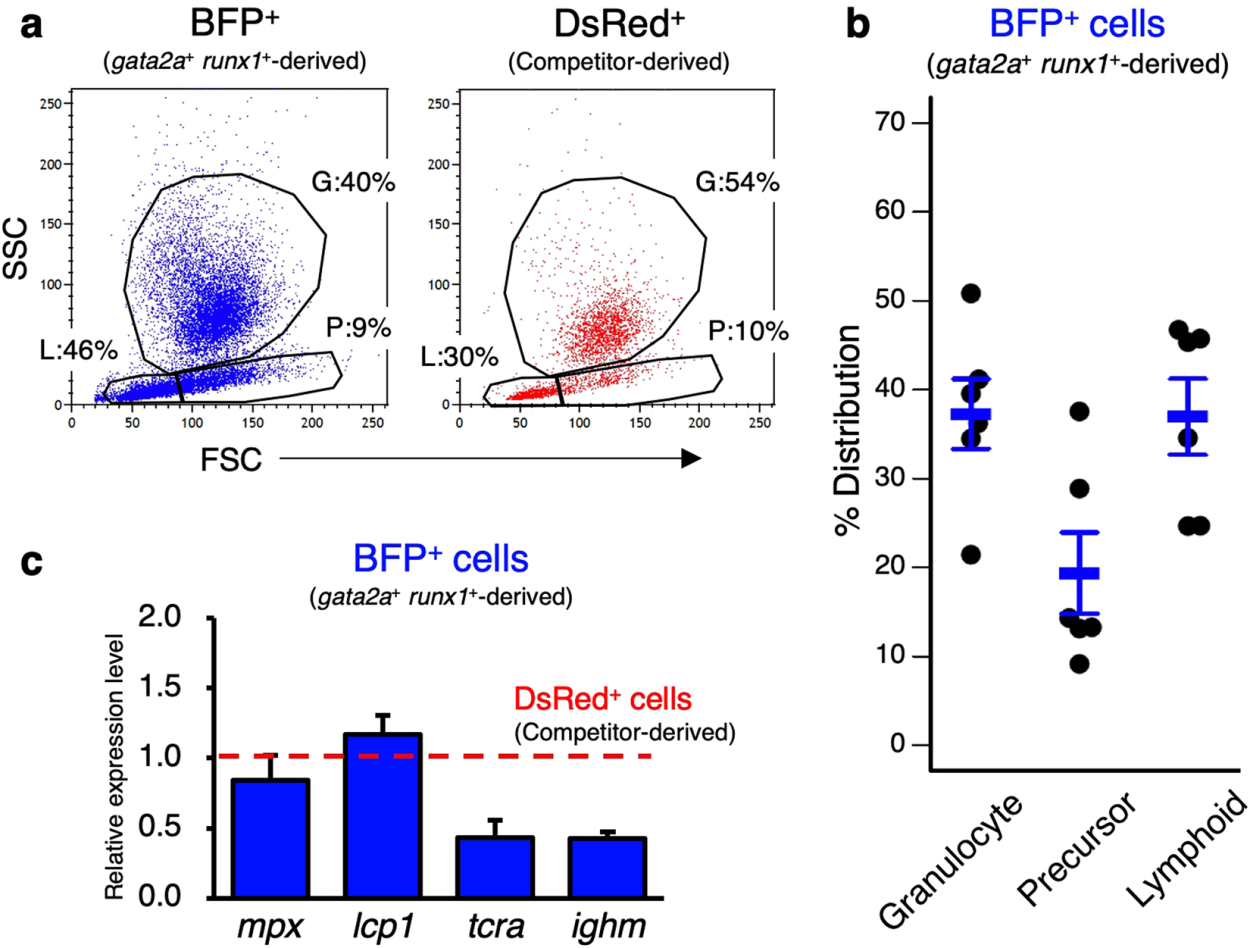
Multilineage differentiation of *gata2a*^+^ *runx1*^+^ cells. (**a**) Representative result of FCM analysis of KMCs in a recipient co-transplanted with BFP-labeled *gata2a*^+^ *runx1*^+^ cells and DsRed-labeled competitors. G, granulocyte; P, precursor; L, lymphoid. (**b**) Percent distribution of *gata2a*^+^ *runx1*^+^-derived BFP^+^ cells in “granulocyte”, “precursor”, and “lymphoid”. Data show the mean ± s.e.m. in the recipients (n = 6). (**c**) Relative expression level of lineage marker genes in isolated *gata2a*^+^ *runx1*^+^-derived BFP^+^ cells in a recipient. Red dotted line indicates the expression level of each gene in competitor-derived DsRed^+^ cells.

HSC potential can be evaluated via an *in vivo* competitive repopulation assay, in which contributions of donor- and competitor-derived cells are compared in an irradiated recipient^4^. To determine if HSCs are enriched in the *gata2a*^+^ *runx1*^+^ fraction, we performed an *in vivo* competitive repopulation assay using a triple transgenic zebrafish, *gata2a:GFP*; *runx1:mCherry*; *bactin2:BFP*, which labels whole blood cells with BFP with the exception of mature erythrocytes where *bactin2* promoter is not active^31^. We also utilized a double transgenic animal, *kdrl:Cre*; *bactin2:loxP-STOP-loxP-DsRed* (“*kdrl-sw*”), as a competitor. As has been previously demonstrated^31^, embryonic expression of the Cre recombinase in the shared vascular precursors of HSCs via the *kdrl* regulatory elements results in nearly all adult leukocytes becoming labeled with DsRed. One hundred BFP-labeled *gata2a*^+^ *runx1*^+^, *gata2a*^−^ *runx1*^+^, and *gata2a*^+^ *runx1*^−^ cells were separately sorted and co-transplanted together with 50,000 DsRed-labeled KMCs (competitor) into sublethally irradiated recipients. At 16 weeks post-transplantation (wpt), donor chimerism was determined by the percentage of BFP^+^ cells within the total BFP^+^ (donor-derived) and DsRed^+^ (competitor-derived) cells in each recipient kidney (Fig. 4a). Although competitor-derived DsRed^+^ cells were detected in all surviving recipients, BFP^+^ donor-derived cells were detected only in recipients transplanted with *gata2a*^+^ *runx1*^+^ cells with the exception of one recipient transplanted with *gata2a*^+^ *runx1*^−^ cells. The mean percentage of BFP^+^ cells within total BFP^+^ and DsRed^+^ cells was 51.9 ± 13.5% (n = 9, ±s.e.m) in recipients transplanted with *gata2a*^+^ *runx1*^+^ cells (Fig. 4b,c). BFP^+^ cells derived from *gata2a*^+^ *runx1*^+^ cells were resolved in three distinct blood cell populations, “granulocyte”, “precursor” and “lymphoid”, with the similar ratio to competitor-derived DsRed^+^ cells (Fig. 5a,b). Moreover, lineage marker genes including *mpx* (granulocyte marker), *lcp1* (macrophage marker), *tcra* (T cell marker), and *ighm* (B cell marker) were detected in isolated donor-derived BFP^+^ cells as well as competitor-derived DsRed^+^ cells (Fig. 5c), indicating that *gata2a*^+^ *runx1*^+^ cells possess the ability of long-term and multipotent hematopoietic reconstitution. Based on the contribution of BFP^+^ cells to DsRed^+^ cells, the frequency of HSCs in *gata2a*^+^ *runx1*^+^ cells was estimated to be approximately 540 times higher than that in KMCs, which is close to the frequency of *gata2a*^+^ *runx1*^+^ cells in KMCs (approximately 1/625). Collectively, these data suggest that most long-term repopulating HSCs are present in the *gata2a*^+^ *runx1*^+^ fraction in the zebrafish kidney.

### *gata2a*^−^*runx1*^+^ cells abundantly contain hematopoietic progenitor cells

**Figure 6:**
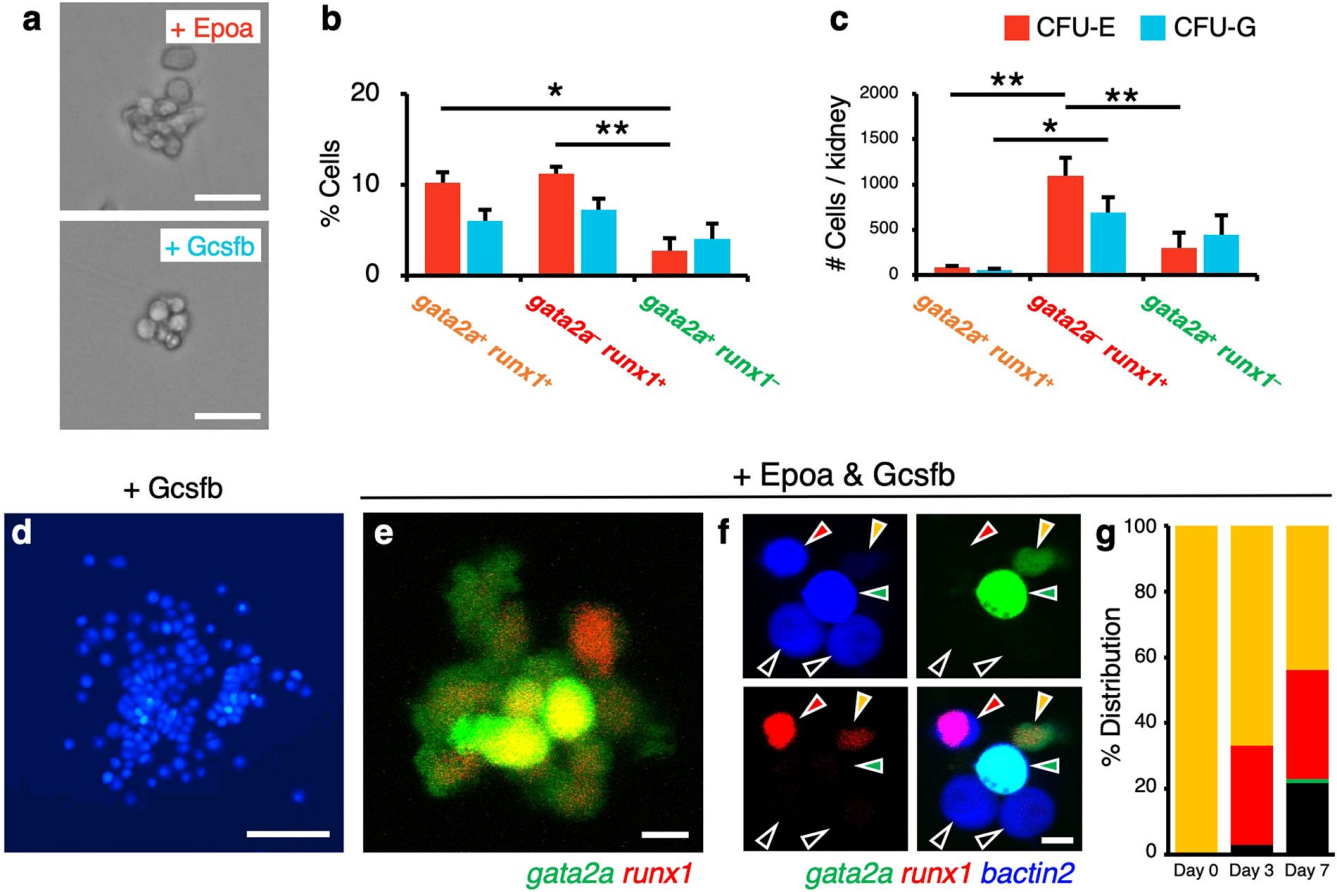
Colony-forming assays of HSPC populations. (**a**) Representative images of a colony formed in the presence of Epoa (CFU-E) or Gcsfb (CFU-G). (**b**,**c**) Percentage (**b**) and absolute number (**c**) of CFU-E (red bars) and CFU-G (blue bars) in *gata2a*^+^ *runx1*^+^, *gata2a*^−^ *runx1*^+^, or *gata2a*^+^ *runx1*^−^ cells in the kidney. Data are shown as mean ± s.e.m. *p < 0.01; **p < 0.001 by one-way ANOVA with Dunnett’s test. (**d**) Representative image of a large colony observed in the well plated with *gata2a*^+^ *runx1*^+^ cells in the presence of Gcsfb. The image shows cells expressing BFP. (**e**,**f**) Representative images of a colony formed by *gata2a*^+^ *runx1*^+^ cells in the presence of both Epoa and Gcsfb. Orange, red, green, and black arrowheads in (**f)** indicate *gata2a*^+^ *runx1*^+^, *gata2a*^−^ *runx1*^+^, *gata2a*^+^ *runx1*^−^, and *gata2a*^−^ *runx1*^−^ cells, respectively. (**g**) Percent distribution of individual GFP^+^ mCherry^+^ (orange bar), GFP^−^ mCherry^+^ (red bar), GFP^+^ mCherry^−^ (green bar), and GFP^−^ mCherry^−^ (black bar) cells at day 0, 3, and 7 of culture. Bars, 20 μm (**a**), 100 μm (**d**), 5 μm (**e**,**f**).

HSCs produce a heterogenous pool of HPCs, including multipotent progenitors (MPPs). The potential of HPCs can be evaluated by an *in vitro* colony-forming assay. To examine the frequency of HPCs in each hematopoietic subset, we performed colony-forming assays, which can determine the percentage of colony-forming unit-erythroid (CFU-E) and -granulocyte (CFU-G). One hundred BFP-labeled *gata2a*^+^ *runx1*^+^, *gata2a*^−^ *runx1*^+^, and *gata2a*^+^ *runx1*^−^ cells were separately sorted and plated in conditioned media containing Erythropoietin a (Epoa) or Granulocyte colony stimulating factor b (Gcsfb). After 7 days of culture, the percentage of CFU-E and CFU-G in each subset was calculated based on the number of colonies formed. We detected both CFU-E and CFU-G from the wells plated with each hematopoietic subset (Fig. 6a). The percentage of CFU-E in *gata2a*^−^ *runx1*^+^ cells was approximately 4 times higher than that in *gata2a*^+^ *runx1*^−^ cells, whereas there were no significant differences in the percentage of CFU-E and CFU-G between *gata2a*^−^ *runx1*^+^ cells and *gata2a*^+^ *runx1*^+^ cells (Fig. 6b). We also calculated the absolute number of CFU-E and CFU-G based on the percentage of CFUs and the frequency of each hematopoietic subset in the kidney. Due to the higher frequency of *gata2a*^–^ *runx1*^+^ cells in the kidney (Fig. 1b), the absolute number of CFU-E and CFU-G in *gata2a*^–^ *runx1*^+^ cells was approximately 13.6 and 14.0 times higher than that in *gata2a*^+^ *runx1*^+^ cells, respectively (Fig. 6c). These results suggest that *gata2a*^–^ *runx1*^+^ cells abundantly contain erythroid and/or myeloid-primed progenitors in the kidney. It should be noted that very large colonies were observed only in the wells plated with *gata2a*^+^ *runx1*^+^ cells (Fig. 6d), suggesting that actively proliferating MPPs may also be present in the *gata2a*^+^ *runx1*^+^ fraction.

To clarify if *gata2a:GFP* or *runx1:mCherry* expression downregulates during erythroid/myeloid differentiation, *gata2a*^+^ *runx1*^+^ cells were cultured in the presence of both Epoa and Gcsfb and were monitored for GFP and mCherry expression. Although some cells still retained to express both GFP and mCherry (Fig. 6e), there were many mCherry single-positive and double-negative (GFP^−^ mCherry^−^) cells, but only a few GFP single-positive cells within the colonies at 7 days of culture (Fig. 6f). The percentage of mCherry single-positive cells and double-negative cells increased during the culture (Fig. 6g). These results suggest that erythroid/myeloid differentiation leads to downregulation of *gata2a:GFP* and/or *runx1:mCherry*.

**Figure 7:**
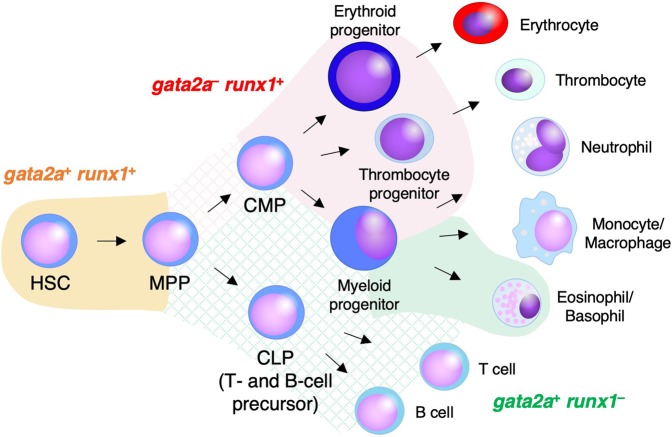
Hematopoietic differentiation in the zebrafish kidney. Schematic diagram of hematopoietic differentiation in the zebrafish kidney is shown. The orange, red, and green area denote the phenotypic transgene expression of *gata2a:GFP* and *runx1:mCherry*. Cross-hatched area shows the putative expression of *gata2a:GFP* and *runx1:mCherry* in hematopoietic progenitors. Long-term repopulating HSCs show *gata2a*^+^ *runx1*^+^. In contrast, *gata2a*^−^ *runx1*^+^ cells are mostly erythroid- and thrombocyte-primed progenitors, but also some myeloid-primed progenitors. Additionally, some myeloid-primed progenitors are *gata2a*^+^ *runx1*^−^. Lymphoid-primed progenitors/precursors may express *gata2a:GFP*, but not *runx1:mCherry*.

Taken together, our transcriptome and functional data suggest that long-term repopulating HSCs are enriched in the *gata2a*^+^ *runx1*^+^ fraction in the kidney. In addition, it is likely that common myeloid progenitors (CMPs) to erythroid-, thrombocyte-, and myeloid-primed progenitors appear to be present mainly within the *gata2a*^−^ *runx1*^+^ fraction. Moreover, common lymphoid progenitors (CLPs) to T- and B-cell precursors and some myeloid progenitors may be present in the *gata2a*^+^ *runx1*^−^ fraction (Fig. 7).

## Discussion

In the present study, we have established a method to isolate HSPCs from the zebrafish kidney utilizing *gata2a:GFP*; *runx1:mCherry* double transgenic animals. This new method will allow us to further investigate the molecular and cellular mechanisms underlying the regulation of HSPCs in the zebrafish kidney.

In mice and humans, hematopoietic cells and mature blood cells can be isolated by a combination of multiple antibodies against cell-surface markers. Due to the lack of antibodies in zebrafish, fluorescent transgenic lines that label specific blood cell types have instead been developed. It is currently possible to isolate various types of blood cells using these transgenic lines, such as erythrocytes (*gata1:DsRed*)^17^, thrombocytes (*cd41:GFP*)^32^, neutrophils (*mpx:GFP*)^33^, eosinophils/basophils (*gata2a:GFP*)^28^, monocytes/macrophages (*mpeg1:GFP*)^34^, T cells (*lck:GFP*)^35^, and B cells (*rag2:GFP*)^36^. To date, however, transgenic lines that label HSPCs are very limited in zebrafish. The mouse *Runx1* + 23 enhancer has been shown to be driven in the HSC population throughout embryonic development, as well as in the adult bone marrow^37^. This stem cell enhancer is also active in zebrafish HSCs and erythroid progenitors^25,38^, whereas its expression does not completely recapitulate endogenous *runx1* expression^25,37^. Because *runx1:mCherry* expression is restricted in hematopoietic cells, this line can be used for imaging of HSPCs not only in embryos but also in juvenile animals^25,39,40^. As a parallel view, *myb:GFP* and *cd41:GFP* are also widely utilized to visualize developing HSPCs in zebrafish embryos^27,31^, while these lines are usually combined with an endothelial mCherry line to capture nascent HSCs derived from hemogenic endothelium. While *myb:GFP* is not shown, *cd41:GFP* can also label HSCs in the adult kidney^24^. *Cd41* is, however, expressed broadly in erythroid, myeloid, and megakaryocyte/thrombocyte lineages in both mammals and zebrafish^32,41,42^. Indeed, our transcriptome data also showed that *cd41* (*itga2b*) is highly expressed in *gata2a*^−^ *runx1*^+^ erythroid and/or myeloid-primed progenitors. Because of these similar expression patterns, we did not utilize a combination of *cd41:GFP* and *runx1:mCherry* to isolate HSCs. In contrast, we found that *gata2a:GFP* expression in the hematopoietic cell fraction was restricted mainly in the lymphoid lineage, a part of the myeloid lineage, and HSCs in the kidney. Thus, this minimum lineage overlapping between *gata2a:GFP* and *runx1:mCherry* enables HSCs to be isolated to the highest degree of purity to date.

Our competitive repopulation assays suggest that the frequency of HSCs is approximately 540 times higher in *gata2a*^+^ *runx1*^+^ cells than in KMCs. The frequency of HSCs in the zebrafish kidney has been estimated by two groups via limiting dilution transplantation assays; they showed that HSCs are present at approximately 1 in 65,500 cells^43^ or 1 in 38,140 cells^44^ within WKMCs (including erythrocytes). Since WKMCs contain approximately 55% of erythrocytes, these observations suggest that the frequency of HSCs in the *gata2a*^+^ *runx1*^+^ fraction is estimated at 1 in 32 to 55 cells. On the other hand, Tamplin *et al*. also estimated by limiting dilution transplantation assays that the frequency of HSCs in the *runx1:mCherry*^+^ fraction of the kidney is at approximately 1 in 35 cells^25^. Our data showed that the percentage of *gata2a*^+^ *runx1*^+^ cells within the *runx1:mCherry*^+^ fraction was approximately 4.6%, and that *gata2a*^−^ *runx1*^+^ cells never showed long-term hematopoietic reconstitution. Based on these observations, the frequency of HSCs in the *gata2a*^+^ *runx1*^+^ fraction could also be estimated at approximately 1 in 1.6 cells. Due to the lack of inbred strains, however, contributions of donor-derived cells following transplantation largely vary in zebrafish, reflecting the difficulty in precise estimation of the HSC frequency by limiting dilution transplantation assay. As one point of view, the percentage of *gata2a*^+^ *runx1*^+^ cells in the zebrafish kidney (0.16% in KMCs) is close to that of c-kit^+^ Sca-1^+^ Lineage-marker^−^ (KSL) cells in the murine bone marrow, which are observed at 0.1–0.2% in total nucleated cells^5,45^. These observations suggest that *gata2a*^+^ *runx1*^+^ cells in the zebrafish kidney may be equivalent to KSL cells in the murine bone marrow.

It has been shown in zebrafish that long-term repopulating HSCs are present in the FSC^low^ SSC^low^ “lymphoid” fraction in the adult kidney^17^. Based on our analysis, however, most *gata2a*^+^ *runx1*^+^ cells were detected in the FSC^mid^ fraction rather than FSC^low^ fraction, whereas both *gata2a*^−^ *runx1*^+^ cells and *gata2a*^+^ *runx1*^−^ cells abundantly contain FSC^low^ cells. Ma *et al*. previously reported the putative HSC fraction, *cd41:GFP*^low^ SP cells, which are detected at approximately 0.02% in WKMCs^24^. Interestingly, these *cd41:GFP*^low^ SP cells also show slightly higher intensity of FSC than *cd41:GFP*^−^ SP cells. In addition, murine HSCs in the bone marrow are larger in cell diameter compared with mature lymphocytes^46^. These observations suggest that HSCs in zebrafish are slightly larger in size compared with FSC^low^ “lymphoid” cells in the kidney.

High-throughput genome-wide transcriptome analysis is now commonly used in all fields of life science research and is promising to characterize cell types along differentiation processes. Tang *et al*. reported single-cell transcriptomic profiling of wide variety of blood cell types isolated from the zebrafish kidney. This analysis revealed that transcriptional programs in predicted blood cell types, including HSPCs, erythroid cells, neutrophils, thrombocytes, T cells, B cells, and NK cells, are highly consistent with those in each blood cell subset reported in mouse and human, suggesting that hematopoietic differentiation programs are highly conserved amongst vertebrates^38^. Our data also showed that *gata2a*^+^ *runx1*^+^ cells highly expressed HSC-related genes, such as *gata2b*, *runx1t1*, *kita*, *mpl*, *gfi1aa*, *meis1b*, *egr1*, *fgd5b*, *pbx1b*, *fosb*, *myca*, and *apoeb*, of which orthologues are shown to be predominantly expressed in mammalian HSCs^47–50^. These data strongly suggest that regulatory mechanisms underlying self-renewal and differentiation of HSCs are also highly conserved amongst vertebrates. Furthermore, the recent advent of direct gene knockout methods in zebrafish based on the CRISPR/Cas9 system enables to rapidly screen the function of candidate genes in the embryonic to adult stage^14–16^. In combination with a *gata2a:GFP* and *runx1:mCherry* line, it is now possible to perform rapid genome-wide interrogation of gene function in HSCs using the zebrafish model. Thus, our purification strategy of HSCs in the zebrafish kidney will open new avenues to elucidate molecular cues that needed to regulate HSCs.

## Methods

### Zebrafish husbandry

Zebrafish strains, AB*, *Tg(gata2a:GFP)*^*la3*^ (ref.^28^), *Tg(Mmu.Runx1:NLS-mCherry)*^*cz2010*^ (here denoted as *runx1:mCherry*) (ref.^25^), *Tg(bactin2:loxP-BFP-loxP-DsRed)*^*sd27*^ (here denoted as *bactin2:BFP*) (ref.^51^), *Tg(kdrl:Cre)*^*s898*^ (ref.^31^), and *Tg(bactin2:loxP-STOP-loxP-DsRed)*^*sd5*^ (ref.^31^), were raised in a circulating aquarium system (AQUA) at 28.5 °C in a 14/10 h light/dark cycle and maintained according to standard protocols^52^. All experiments were performed in accordance with a protocol approved by the Committee on Animal Experimentation of Kanazawa University.

### Cell preparation and flow cytometry

Kidney marrow cells (KMCs) were prepared as previously described^51^ with some modifications. Cells were obtained by pipetting of a dissected kidney in 1 mL of ice-cold 2% fetal bovine serum (FBS) in phosphate buffered saline (PBS) (2% FBS/PBS). After centrifugation, the pellet was gently mixed with 1 mL of distilled water by pipetting to lyse erythrocytes by osmotic shock. Subsequently, 1 mL of 2X PBS was added. Cells were then filtered through a 40-stainless mesh and washed with 2% FBS/PBS by centrifugation. Just before flow cytometric analysis, the Sytox Red (Thermo Fisher Scientific) was added at a concentration of 5 nM to exclude dead cells. Flow cytometric acquisition and cell sorting were performed on a FACS Aria III (BD Biosciences). Data analysis was performed using the Kaluza software (ver. 1.3, Beckman Coulter). The absolute number of cells was calculated by flow cytometry based on the acquisition events, maximum acquisition times, and the percentage of each cell fraction.

### RNA-seq and qPCR

For sorted cells, whole-transcript amplification and double-strand cDNA synthesis was performed according to the method of Quartz-Seq^53^. Cells were directly sorted in a lysis buffer containing 1 μg/mL of polyinosinic-polycytidylic acid, and total RNA was extracted using RNeasy Mini Kit (Qiagen). Reverse transcription (RT) was performed using Super Script III (Thermo Fisher Scientific) and an RT primer, which contains oligo-dT, T7 promoter, and PCR target region sequences. After digestion of remaining RT primers by exonuclease I (Takara), a poly-A tail was added to the 3′ ends of the first-strand cDNAs using terminal transferase (Sigma). The second-strand DNA was then synthesized using MightyAmp DNA polymerase (Thermo Fisher Scientific) and a tagging primer, which contains oligo-dT and PCR target region sequences. PCR amplification was performed using a suppression primer, which allow to amplify small-size DNA that contains complementary sequences at both ends of the template DNA. The amplified double-strand cDNA was purified using QIAquick PCR Purification Kit (Qiagen). Library preparation was performed using Nextera XT DNA Library Preparation Kit (illumina). Next generation sequencing of cDNA libraries was performed by GENEWIZ using the Illumina NextSeq. 500 (illumina), and base-calling was performed using the Illumina RTA software (ver. 2.4.11). Sequence reads were mapped to the zebrafish reference genome (GRCz11) using HiSAT2 (version 2.1.0). Reads per million (RPM) were calculated using the Subread (ver. 1.6.4). Hierarchical clustering of each subset was performed in R (ver. 3.5.0) with the Bioconductor Heatplus package. Genes that were over two-fold enriched in *gata2a*^+^ *runx1*^+^ cells, *gata2a*^−^ *runx1*^+^ cells, or *gata2a*^+^ *runx1*^−^ cells were selected and used for gene ontology enrichment analysis using the Gene Ontology Resource website (http://geneontology.org). The data have been deposited in Gene Expression Omnibus (GEO) (National Center for Biotechnology Information) and are accessible through the GEO database (series accession number, GSE132927). For the kidney tissue, total RNA was extracted using RNeasy Mini Kit, and cDNA was synthesized using ReverTra Ace qPCR RT Master Mix (Toyobo). Quantitative real-time PCR (qPCR) assays were performed using TB Green Premix Ex Taq II (TaKaRa) on a ViiA 7 Real-Time PCR System according to manufacturer’s instructions (Thermo Fisher Scientific). Primers used for qPCR were listed in Supplementary Table S2. The expression of *ef1a* was used for normalization.

### X-ray irradiation and transplantation

In zebrafish, sublethal dose of γ-irradiation (23–25 Gy) has been shown to be sufficient to ablate hematopoietic cells in the adult kidney^18,43^. Three to six zebrafish were placed in a 90 mm petri dish in system water, and animals were sublethally irradiated with X-ray on a Faxitron RX-650 (Faxitron, 130 kVp, 1.15 Gy/min) for 20 min (approximately 23 Gy). At 2 days post-irradiation, animals were transplanted with cells using a retro-orbital injection method^43^.

### Colony assay

Recombinant zebrafish Epoa and Gcsfb (also known as colony stimulating factor 3b (Csf3b)) proteins were generated as previously described^54^. The coding region of *epoa* and *gcsfb* was amplified by PCR using kidney cDNA and primers listed in Supplementary Table S2, and ligated into the *pET-16b* vector. Recombinant Epoa and Gcsfb were purified from *Escherichia coli* using QIAexpressionist kit (Qiagen) according to the manufacturer’s procedure. For colony assays, cells were sorted into a round-bottom 96-well plate or a glass-bottom of 35 mm dish filled with the 1X ERDF condition medium containing 20% FBS, 2.5% carp serum, and 600 ng/mL of Epoa and/or Gcsfb. Cells were cultured at 30 °C, 5% CO_2_ for 7 days. Colonies were enumerated using an EVOS fl microscope (Thermo Fisher Scientific).

### Statistical analysis

Statistical differences between groups were determined by one-way ANOVA with Dunnett’s test or Fisher’s Exact test. A value of *p* < 0.05 was considered to be statistically significant.

## Supplementary information

Supplementary information
