# Supplementary material for: Enrichment of hematopoietic stem/progenitor cells in the zebrafish kidney
Source: Sci Rep. 2019 Oct 2;9:14205. doi: 10.1038/s41598-019-50672-5 (PMC6775131; doi:10.1038/s41598-019-50672-5)
Supplement: Supplementary file 1 — Supplementary information [file 41598_2019_50672_MOESM1_ESM.pdf]

Supplementary information file

Enrichment of hematopoietic stem/progenitor cells in the zebrafish kidney

Isao Kobayashi[1], Mao Kondo[2], Shiori Yamamori[2], Jingjing Kobayashi-Sun[2], Makoto Taniguchi[3], Kaori Kanemaru[4], Fumihiko Katakura[5], and David Traver[6]

[1]Faculty of Biological Science and Technology, Institute of Science and Engineering, Kanazawa University, Ishikawa, Japan

[2]Division of Life Sciences, Graduate School of Natural Science and Technology, Kanazawa University, Ishikawa, Japan

[3]Department of Life Science, Medical Research Institute, Kanazawa Medical University, Ishikawa, Japan

[4]Department of Applied Biological Science, Faculty of Science and Technology, Tokyo University of Science, Chiba, Japan

[5]Laboratory of Comparative Immunology, Department of Veterinary Medicine, Nihon University, Kanagawa, Japan

[6]Department of Cellular and Molecular Medicine, University of California at San Diego, La Jolla, CA, USA

Supplementary Table S1. Expression levels of selected genes

Ensembl gene ID	gata2a+ runx1+	gata2a− runx1+	gata2a+ runx1−	Gene symbol	Description
HSPC markers					
ENSDARG00000100075	6.03	0.73	13.10	*abcg2a*	ATP-binding cassette, sub-family G (WHITE), member 2a
ENSDARG00000040295	1.11	0.00	0.00	*apoeb*	apolipoprotein Eb
ENSDARG00000041959	1251.29	412.03	1248.60	*cxcr4b*	chemokine (C-X-C motif), receptor 4b
ENSDARG00000037421	474.79	94.81	212.24	*egr1*	early growth response 1
ENSDARG00000017320	1.85	0.49	0.25	*f11r.1*	F11 receptor, tandem duplicate 1
ENSDARG00000089622	0.49	0.00	0.00	*fgd5b*	FYVE, RhoGEF and PH domain containing 5b
ENSDARG00000055751	37.30	10.60	10.50	*fosb*	FBJ murine osteosarcoma viral oncogene homolog B
ENSDARG00000059327	344.43	11.82	1354.10	*gata2a*	GATA binding protein 2a
ENSDARG00000009094	143.29	17.18	10.99	*gata2b*	GATA binding protein 2b
ENSDARG00000020746	52.19	1.83	1.98	*gfi1aa*	growth factor independent 1A transcription repressor a
ENSDARG00000013539	506.05	224.60	482.17	*ikzf1*	IKAROS family zinc finger 1 (Ikaros)
ENSDARG00000043317	8.12	4.75	0.25	*kita*	KIT proto-oncogene, receptor tyrosine kinase a
ENSDARG00000012078	461.37	209.61	8.28	*meis1b*	Meis homeobox 1 b
ENSDARG00000039222	5.91	2.92	0.25	*mpl*	MPL proto-oncogene, thrombopoietin receptor
ENSDARG00000053666	78.54	70.19	28.78	*myb*	v-myb avian myeloblastosis viral oncogene homolog
ENSDARG00000045695	380.86	161.59	161.96	*myca*	MYC proto-oncogene, bHLH transcription factor a
ENSDARG00000101131	2.09	0.24	0.00	*pbx1b*	pre-B-cell leukemia homeobox 1b
ENSDARG00000087646	4.80	3.53	2.35	*runx1*	runt-related transcription factor 1
ENSDARG00000003680	84.32	26.20	5.81	*runx1t1*	runt-related transcription factor 1; translocated to, 1
ENSDARG00000004588	240.78	407.88	55.47	*sox4a*	SRY (sex determining region Y)-box 4a
ENSDARG00000019930	457.06	1187.33	61.89	*tal1*	T-cell acute lymphocytic leukemia 1
ENSDARG00000041572	77.06	73.85	11.12	*zfpm1*	zinc finger protein, FOG family member 1
Erythroid-myeloid markers					
ENSDARG00000013477	4.92	39.97	0.00	*gata1a*	GATA binding protein 1a
ENSDARG00000097011	5.42	61.42	0.62	*hbaa1*	hemoglobin, alpha adult 1
ENSDARG00000018687	37.79	367.54	0.37	*itga2b*	integrin, alpha 2b
ENSDARG00000017400	14.16	159.76	0.00	*klf1*	Kruppel-like factor 1 (erythroid)
ENSDARG00000038792	29.17	146.73	0.25	*klf17*	Kruppel-like factor 17
ENSDARG00000023188	2462.57	999.29	3648.33	*lcp1*	lymphocyte cytosolic protein 1 (L-plastin)
ENSDARG00000095019	170.74	201.20	56.58	*lmo2*	LIM domain only 2 (rhombotin-like 1)

ENSDARG00000057789	0.12	0.49	0.62	*lyz*	lysozyme
ENSDARG00000019521	105.86	214.12	50.28	*mpx*	myeloid-specific peroxidase
ENSDARG00000000767	55.76	91.40	516.14	*spi1b*	Spi-1 proto-oncogene b
Lymphoid markers					
ENSDARG00000070668	0.00	0.00	0.25	*cd4-1*	CD4-1 molecule
ENSDARG00000044797	0.00	0.00	0.86	*cd8a*	CD8a molecule
ENSDARG00000016526	34.34	18.52	369.38	*gata3*	GATA binding protein 3
ENSDARG00000096280	0.00	0.00	138.12	*ighz*	immunoglobulin heavy constant zeta
ENSDARG00000061083	0.25	0.00	208.29	*igl4v8*	immunoglobulin light 4 variable 8
ENSDARG00000102525	0.00	0.00	0.25	*lck*	LCK proto-oncogene, Src family tyrosine kinase
ENSDARG00000079105	0.00	0.00	0.00	*mhc2dab*	major histocompatibility complex class II DAB gene
ENSDARG00000056330	0.00	0.00	0.00	*mhc2dbb*	major histocompatibility complex class II DBB gene
ENSDARG00000037383	0.00	0.00	31.87	*pax5*	paired box 5
ENSDARG00000052122	0.00	0.00	3.95	*rag1*	recombination activating gene 1
ENSDARG00000111519	0.00	0.00	0.00	*traj15*	T-cell receptor alpha joining 15
ENSDARG00000099028	0.00	0.00	0.00	*trdj2*	T-cell receptor delta joining 2

Supplementary Table S2. Primer sequences

Gene	Forward primer	Reverse primer	Description
gata2b	GACTGGGACCACCACACTCT	AAGGAGGATGGTTTGTCGTG	qPCR
gfi1aa	TTCTGGAGACCCCTGTCATC	TGCAGGACTGAATGAGATGC	qPCR
kita	ACCCTGTCAAGAGACCCTCA	AGCCGATGGACATGAGAACT	qPCR
myb	TGATGCTTCCCAACACAGAG	TTCAGAGGGAATCGTCTGCT	qPCR
gata1a	ATTATTCCACCAGCGTCCAG	GGACGTGGAGGTGTGAGAGT	qPCR
mpl	GGTCCTGGAAAACATCCTGA	TGCTCAGAGAGCAGAGGACA	qPCR
itga2b	TGATGAATGTGCCCTCAAAA	GCAGCCAATAGAAGCAGTCC	qPCR
mpx	CTTTTGATCTCGAGCCTTGG	CTTTAGCAGTGGCAGGAAGG	qPCR
lcp1	GGCATACGGGAGAAAGATGA	TGAAGGTCCGCTCCTCTCTA	qPCR
lck	GAGACCTGAGAGCTGCCAAT	GCCGTAGTTTATGGCCTCTG	qPCR
tcra	TCGTTTTCAATGTGCTGGTG	GATGATCTGGAATGGGATGC	qPCR
ighm	GGAAAAACATTGCCAGAGGA	TGTACCCATACGCAGGTTCA	qPCR
rag1	CATTTGCCGAAAAGGAAGAA	ACTGCAGCTGAGGAAGGTGT	qPCR
ef1a	ACCGGCCATCTGATCTACAA	CAATGGTGATACCACGCTCA	qPCR
epoa	CCACATATGTCCTCCCCATTACGCCCCAT	AATGGATCCTCAGCTGACACCCTGTCGAC	Recombinant protein
csf3b	CACCATATGACTGAACACACTCTGAGAGCCTT	GCAGGATCCTTAGCATTGAGACACACTGATGC	Recombinant protein

Primer sequence for whole-transcript amplification	Description
TATAGAATTCGCGGCCGCTCGCGATAATACGACTCACTATAGGGCGTTTTTTTTTTTTTTTTTTTTTTTT	RT primer
TATAGAATTCGCGGCCGCTCGCGATTTTTTTTTTTTTTTTTTTTTTTT	Tagging primer
(5' Aminolink)-GTATAGAATTCGCGGCCGCTCGCGAT	Suppression primer
